# Collaborative artificial intelligence system for investigation of healthcare claims compliance

**DOI:** 10.1038/s41598-024-62665-0

**Published:** 2024-05-24

**Authors:** Marco Luca Sbodio, Vanessa López, Thanh Lam Hoang, Theodora Brisimi, Gabriele Picco, Inge Vejsbjerg, Valentina Rho, Pol Mac Aonghusa, Morten Kristiansen, John Segrave-Daly

**Affiliations:** 1 IBM Research Europe, Building 3, IBM Technology Campus, Damastown Industrial Park, Mulhuddart, Dublin 15, Ireland; 2 IBM Watson Health, Building 3, IBM Technology Campus, Damastown Industrial Park, Mulhuddart, Dublin 15, Ireland

**Keywords:** Health policy, Health care economics

## Abstract

Healthcare fraud, waste and abuse are costly problems that have huge impact on society. Traditional approaches to identify non-compliant claims rely on auditing strategies requiring trained professionals, or on machine learning methods requiring labelled data and possibly lacking interpretability. We present Clais, a collaborative artificial intelligence system for claims analysis. Clais automatically extracts human-interpretable rules from healthcare policy documents (0.72 F1-score), and it enables professionals to edit and validate the extracted rules through an intuitive user interface. Clais executes the rules on claim records to identify non-compliance: on this task Clais significantly outperforms two baseline machine learning models, and its median F1-score is 1.0 (IQR = 0.83 to 1.0) when executing the extracted rules, and 1.0 (IQR = 1.0 to 1.0) when executing the same rules after human curation. Professionals confirm through a user study the usefulness of Clais in making their workflow simpler and more effective.

## Introduction

Healthcare fraud, waste, and abuse (FWA) have a direct impact on society because they divert funds that states could legitimately use to deliver services helping citizens in need. The definitions^1^ of fraud, waste and abuse can be summarized as follows: fraud is a willing attempt to obtain payments from a healthcare benefit program by means of fraudulent pretenses; waste is the overutilization of services that results in unnecessary costs to the healthcare system; and, finally, abuse is a payment obtained without intentionally misrepresenting facts but with no legal entitlement to the payment. A literature review of healthcare waste from 2012 to 2019^2^ estimates that waste-related cost in the US healthcare system ranges from $760 billion to $935 billion, accounting for approximately 25% of total healthcare spending. Federal agencies in the US estimate^3^ that cumulative improper payments were over $2 trillion between 2003 and 2021.

Claims that are not compliant with healthcare policies are a major cause of fraud, waste and abuse, and their detection appears both as a research problem in literature and as a business problem in industry. Countries with insurance-based health programs (such as Medicare and Medicaid in the US) often employ professional investigators to validate the compliance of claims submitted by providers. This is a labor-intensive and costly task: claims volumes are high, and expert investigators are needed to interpret policy documents correctly and to identify the data required to verify compliance. Systems for processing claims have been extended to perform automated analysis on claims data (incomplete and duplicate records, or medical services that are not covered by healthcare policies). Professionals typically support these functions by manually developing rules and translating them into algorithms that execute on claim databases. This process is very costly: each policy document may require many rules, and every time a policy changes, rules may need updates, become obsolete, or new rules may be required; additionally, changes to rules require changes to the corresponding algorithms.

Governments regulate a wide range of sectors with policies, and recently the multidisciplinary initiative known as ‘Rules As Code’^4–7^ proposed creating a machine-consumable version of government rules to complement the existing natural language counterpart. This early-stage initiative has the potential to reduce non-compliance costs through automated checking and to enable a more efficient, and transparent application of government policies. Research on using AI to support rule extraction from policy and regulatory text^8–12^ is still limited, and in general, none of the existing approaches focus on collaboratively supporting policy experts in reducing healthcare FWA or automatically extracting human-interpretable and executable rules to identify non-compliant claims providing explanations of the results.

Conversely, recent research approaches^13–22^ have trained machine learning and deep learning algorithms to identify fraudulent providers using public datasets, and have analyzed different techniques to deal with the class imbalance problem typical of labelled claims datasets (only a very small percentage of claims are labelled as fraudulent). Finally, semantic embeddings ^23^ have been proposed to represent providers’ specialties, which are then used as a predictor for detecting fraud. The use of data-driven machine learning algorithms has far better scalability than manually defined rules, and given enough labelled data, it is possible to train machine learning algorithms to identify suspicious providers. However, data-driven approaches have two issues: they typically require a vast amount of good quality labelled data, which is often not available, and the interpretability of their results with respect to policy documents may be problematic. The interpretability is particularly important for professionals because they need to explicitly relate suspicious claims to some specific text in a policy document to substantiate their investigations.

**Figure 1:**
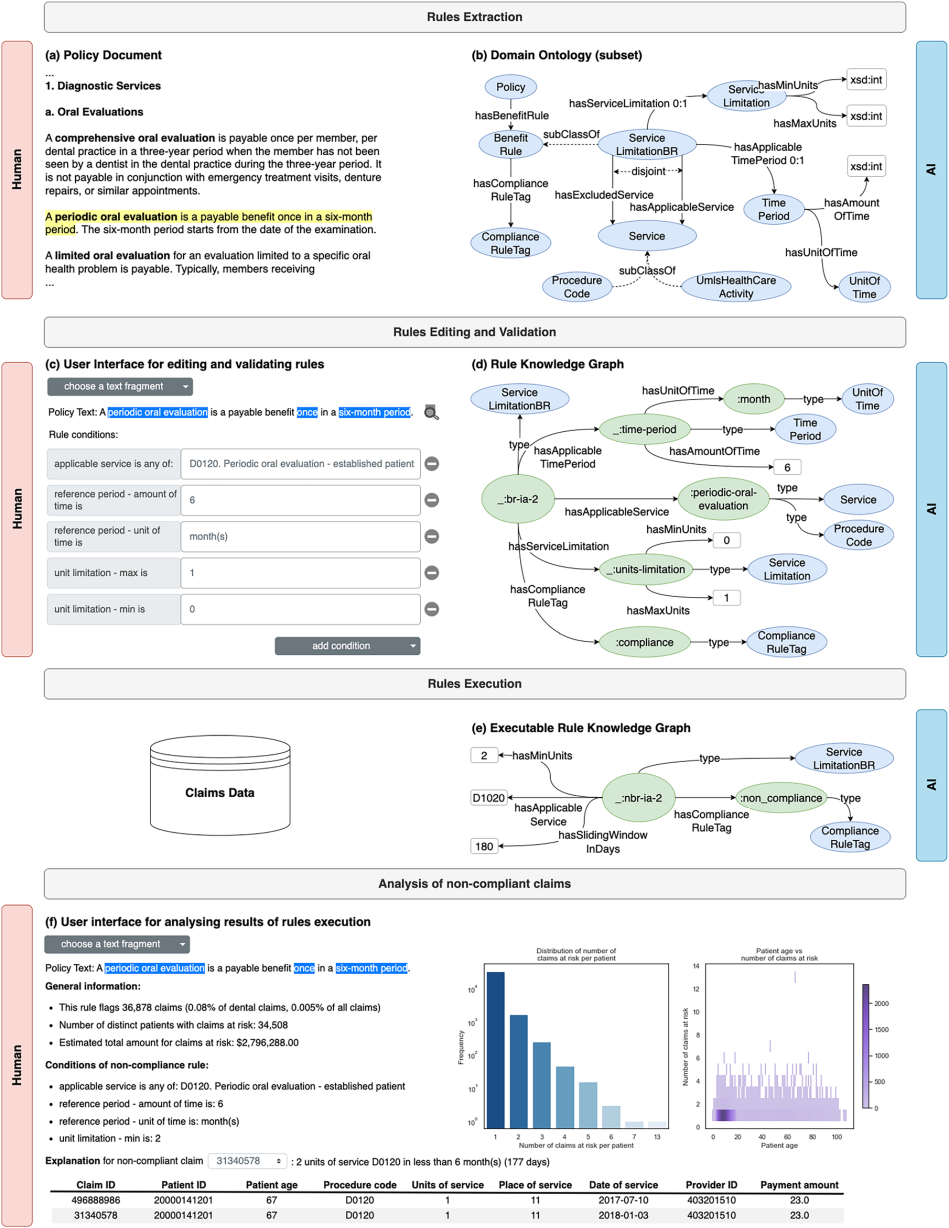
Overview of Clais, a human-AI collaborative system to investigate the compliance of healthcare claims. The system takes as input a policy document (**a**), it identifies fragments of text that potentially contain rules defining compliance (or non-compliance) of claims (see for example the sentence highlighted in yellow). The AI system uses a domain ontology (**b**) to guide the translation of text into a formal rule knowledge graph (**d**). The system visualizes the rule knowledge graph in a user interface (**c**) that displays both the original fragment of text from the policy document and the conditions of the rule in a human-understandable format. Human experts can edit the rule, and finally validate it using the user interface; they can also compose (using a library of conditions) new rules corresponding to fragments of policy text that the system failed to identify. After human validation, the AI system performs a normalization step (**e**) that produces an executable rule knowledge graph. If the original rule is a compliance rule, then the normalization transforms it into a non-compliance rule (by reversing compliance conditions). Finally, the normalization step produces a mapping from the rule conditions (as defined in the ontology) to the fields in claims records, thus making it possible to execute the rule knowledge graph on the claims data to identify claims at risk. The system displays the results of the rule execution in a user interface (**f**), where human experts can see aggregated statistics (for example distribution of the number of claims at risk per patient, distribution of patient age vs number of claims at risk, total number of claims at risk, and their estimated value, etc.). The user interface also shows the details of each claim at risk (in the context of the sequence of claims for the patient), including a human understandable explanation of which values in the claim record violate conditions in the rule.

We developed Clais to tackle the limitations of existing approaches based on manually defined rules or data-driven machine learning algorithms, and to implement a collaborative workflow where professionals and AI cooperate to identify claims that are not compliant with a healthcare policy. Clais (see Fig. 1) analyzes policy documents, identifies paragraphs of text that may define compliance or non-compliance definitions of patients’ benefits and translates them into rules that are both human interpretable and machine executable. We build on our previous work^24–26^ leveraging different natural language processing techniques and a rich domain ontology (co-created with domain experts), which captures repeatable templates for the translation of policy text into rules, even in the absence of labelled data. The rules, formalized as knowledge graphs, consist of a set of logical conditions having precise semantics (defined in the ontology). Clais displays the rules, together with the corresponding paragraphs of text from the policy document, in an intuitive user interface where human experts can easily modify and validate them; they can also interactively build new rules (using a library of conditions automatically derived from the ontology) corresponding to fragments of text that the system failed to identify. After validation, all rules are stored in a shared knowledge store that complements the policy documents with referenceable, human-interpretable, and formal representations of patients’ benefits and eligibility requirements. Finally, Clais executes the validated rules directly on claims data to identify potentially non-compliant claims (referred to as “at-risk”) and presents the results in a visual interface where professionals have access to both aggregated and detailed information on claims at-risk, including explanations on why a claim is non-compliant with a rule, and corresponding evidence from claims data. Such evidence-based view helps investigators in assessing claims at-risk, identifying providers that violate policies, and prioritize their work.

We evaluate Clais using two dental policies from two states in the US. Dental spending by US government programs increased by 25% from 2020 to 2021 ^27^, and policy makers are considering extending dental coverage in Medicare ^28^: these facts make the dental domain interesting for FWA investigations, and our domain experts confirmed that the structure of rules extracted from dental policies is generalizable to other domains. We measure the performance of Clais on two tasks. In the first task we use Clais to extract rules from policies and report its performance with respect to a ground truth consisting of rule definitions manually created by domain experts. In the second task we use Clais to identify non-compliant claims by executing both the automatically extracted rules and the corresponding validated rules on a database of almost 800 million claims containing almost 45 million dental claims. In the second task we also compare Clais to two baseline data-driven models using a ground truth consisting of non-compliant claims labelled using algorithms manually developed by domain experts. Finally, we report the results of a user study conducted with professional FWA investigators confirming the usefulness of our system.

## Results

We evaluated Clais along three dimensions: extraction of rules from policy documents, execution of rules on claim records to identify non-compliance, and perceived usefulness by professional FWA investigators.

For the first two tasks (extraction and execution of rules) we built a ground truth^24,25,29^ with the help of domain experts who manually defined rules corresponding to policy documents for dental providers for the states of Iowa^30^ and Colorado^31^ (US)—all domain experts who helped design and test Clais are professional FWA investigators. The resulting 141 ground truth rules are based on the same ontology that guides Clais automatic extraction of rules from text, and are formalized into knowledge graphs; there are 90 rules from the Iowa policy document (a knowledge graph with 1977 vertices and 2447 edges), and 51 rules from the Colorado policy document (a knowledge graph with 1651 vertices and 2044 edges); the ontology and the ground truth rules are publicly available^32^. The ground truth rules include examples of all rule types that domain experts identified as useful to check claims compliance: 112 service limitation rules (which define constraints on the number of units or the monetary amount a provider can bill for a service per patient over a period of time), 25 mutually exclusive rules (which define disjoint pairs of services that cannot be reimbursed together for the same patient, usually within a time window), and 4 non-coverage rules (which explicitly list services that cannot be reimbursed under certain conditions). Additionally, the domain experts manually developed 20 ground truth algorithms (using their standard development tools and environment) corresponding to 20 ground truth rules. We executed the ground truth algorithms to label claims at-risk from a database $\mathcal{D}$ of 798,181,509 patient claims, 44,913,580 of which (5.63%) are dental claims. The records in the claims database were anonymized and unlabeled; they were extracted from the MarketScan database^33,34^, which contains data from more than 40 contributing health plans and captures data from more than 250 million unique individuals. The 20 ground truth algorithms identified 421,321 positive samples in the database $\mathcal{D}$ (potentially non-compliant claims), which corresponds to 0.94% of the dental claims in the database. We used the 141 ground truth rules to measure the performance of Clais in extracting correct rules from policy documents, and we used the claim records labelled by the 20 ground truth algorithms to measure the performance of Clais in identifying non-compliant claims.

We computed precision, recall and F1 (the harmonic mean of precision and recall) for the extraction task. Given the set $\mathcal{G}$ of ground truth rules and the set $\mathcal{E}$ of automatically extracted rules, we computed precision as $\left|\mathcal{G}{\cap }^{*}\mathcal{E}\right|/\left|\mathcal{E}\right|$ (the fraction of the extracted rules that fully or partially match ground truth rules) and recall as $\left|\mathcal{G}{\cap }^{*}\mathcal{E}\right|/\left|\mathcal{G}\right|$ (the fraction of ground truth rules that are fully or partially extracted). The intersection ${\cap }^{*}$ includes both fully and partially matched rules. An extracted rule ${R}_{x,E}\in \mathcal{E}$ fully or partially matches a ground truth rule ${R}_{x,G}\in \mathcal{G}$ if ${R}_{x,E}$ and ${R}_{x,G}$ originate from the same text fragment in the same document, and there is a non-empty intersection between the set of conditions (and corresponding values) of ${R}_{x,E}$ and the set of conditions (and corresponding values) of ${R}_{x,G}$. We extended the classical definitions of precision and recall in information retrieval^35^ to include partially matched rules because domain experts confirmed their usefulness (it is often simpler to correct problems in a partially correct rule, rather than creating an entirely new one).

Additionally, we defined three similarity metrics (formal definitions in “Formal definition of rule and similarity metrics”) to measure the similarity of an extracted rule ${R}_{x,E}$ to the corresponding ground truth rule ${R}_{x,G}$: structure similarity (which measures the similarity of the logical structure of the two rules as the Sørensen-Dice coefficient^36,37^ of the set of conditions of ${R}_{x,E}$ and the set of conditions of ${R}_{x,G}$); condition similarity (which measures the similarity among the values of each condition in ${R}_{x,E}$ with respect to the values of the corresponding condition in ${R}_{x,G}$); and an overall rule similarity (the arithmetic mean of the structure similarity and the condition similarities for all the conditions in the two rules).

**Figure 2:**
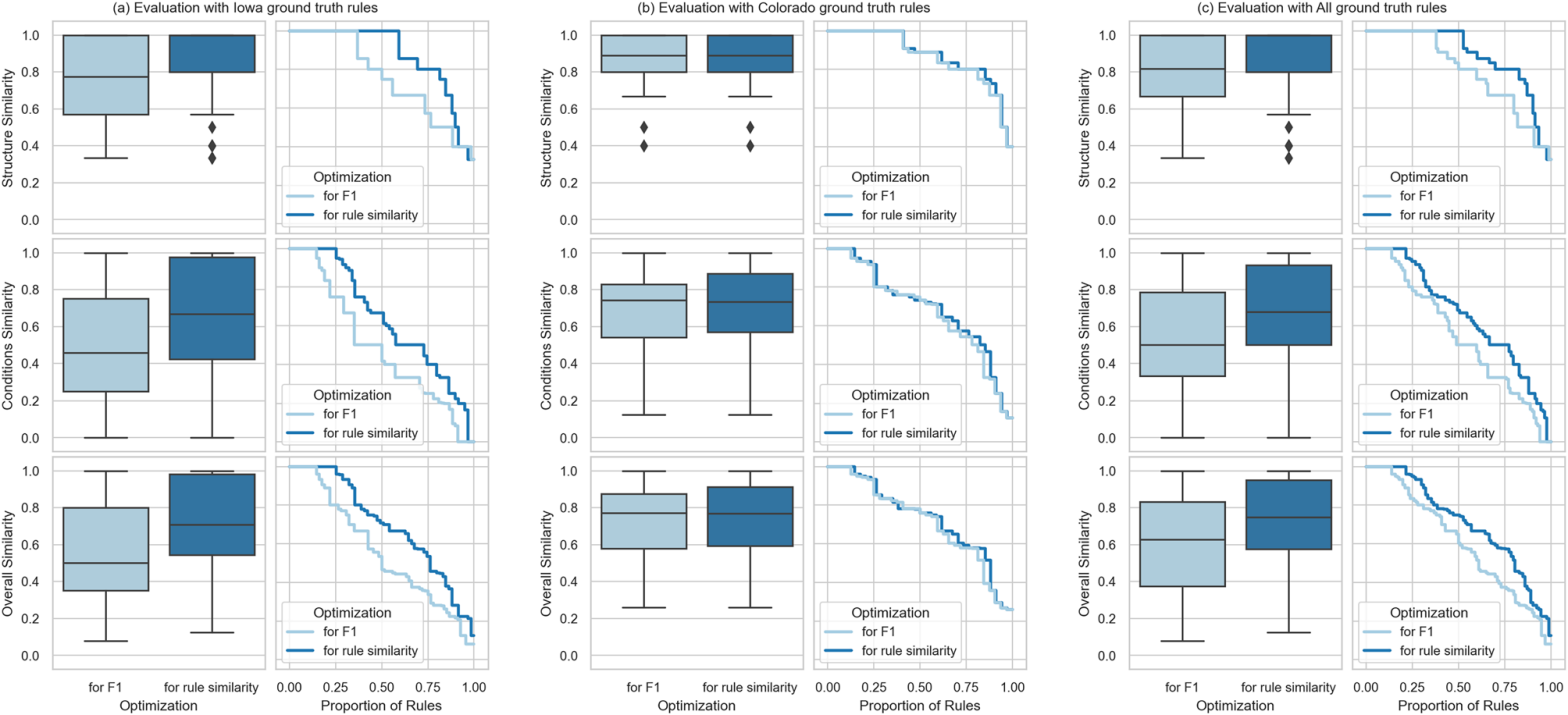
(**a**) The distributions of values (box plots) and the empirical cumulative distribution functions of the rule similarity metrics in the evaluation using the ground truth rules from the Iowa policy document; rows show results for the three rule similarity metrics (structure, conditions, and overall rule similarity) with two configurations of our system (optimized for F1 and optimized for rule similarity). Subfigures (**b,c**) show data from the evaluation using the ground truth rules from the Colorado policy document (**b**), and using all ground truth rules (**c**). We observe that optimizing for rule similarity does not always have significant effects on improving the rule similarity metrics. In the evaluation with the ground truth rules from the Iowa policy document (**a**), the structure similarity and the overall rule similarity obtained when optimizing for rule similarity are significantly greater than the corresponding metrics obtained when optimizing for F1 (the p-value of one-tailed Mann–Whitney test is 0.01824 for the structure similarity and 0.04877 for the overall similarity). In the evaluation with the ground truth rules from the Colorado policy document (**b**) the optimization for rule similarity has no statistically significant effects. Considering all ground truth rules (**c**), the optimization for rule similarity shows statistically significant effects only for the structure similarity metrics (the p-value of one-tailed Mann–Whitney test is 0.03958).

We used hyper-parameter optimization to tune the configurations of the various sub-components of our rule extraction system. Table 1 shows that using a configuration optimized for F1, Clais achieved 0.72 F1-score when evaluated using all ground truth rules. The overall rule similarity was 0.75 (IQR = 0.58 to 0.95) when evaluated using all ground truth rules and a configuration optimized for rule similarity. Figure 2 shows the distributions of values of the rule similarity metrics and their empirical cumulative distribution functions in our evaluations. In our current implementation of Clais (where professionals curate extracted rules before execution on claims data), we used the configuration optimized for F1 because it enabled the system to extract 5% more rules. Although the structure of the rules extracted using this configuration was slightly less accurate (the rule similarity dropped from 0.75 to 0.63), we observed that professionals preferred to use our visual interface (Fig. 1c) to amend partially incorrect rules rather than adding completely new ones.

**Table 1:** Evaluation of automatic extraction of rules.

		Configuration optimized for F1-score	Configuration optimized for rule similarity
Evaluation with ground truth rules from Iowa dental policy document	Precision	0.69	0.66
	Recall	0.76	0.66
	F1	**0.72**	0.66
	Rule structure similarity	0.78 (IQR = 0.57 to 1.00)	1.00 (IQR = 0.80 to 1.00)
	Rule conditions similarity	0.46 (IQR = 0.25 to 0.75)	0.67 (IQR = 0.42 to 0.97)
	Rule overall similarity	0.50 (IQR = 0.35 to 0.80)	**0.71 (IQR = 0.54 to 0.98)**
Evaluation with ground truth rules from Colorado dental policy document	Precision	0.89	0.52
	Recall	0.63	0.67
	F1	**0.74**	0.58
	Rule structure similarity	0.89 (IQR = 0.80 to 1.00)	0.89 (IQR = 0.80 to 1.00)
	Rule conditions similarity	0.74 (IQR = 0.54 to 0.83)	0.73 (IQR = 0.57 to 0.89)
	Rule overall similarity	0.77 (IQR = 0.58 to 0.87)	**0.77 (IQR = 0.59 to 0.91)**
Evaluation with all ground truth rules	Precision	0.74	0.60
	Recall	0.71	0.66
	F1	**0.72**	0.63
	Rule structure similarity	0.82 (IQR = 0.67 to 1.00)	1.00 (IQR = 0.80 to 1.00)
	Rule conditions similarity	0.50 (IQR = 0.33 to 0.79)	0.69 (IQR = 0.50 to 0.93)
	Rule overall similarity	0.63 (IQR = 0.38 to 0.83)	**0.75 (IQR = 0.58 to 0.95)**

The table reports metrics when evaluating Clais with the 90 ground truth rules from the Iowa policy document, the 51 ground truth rules from the Colorado policy document, and all 141 ground truth rules. We report evaluation metrics for two configurations of the extraction system: optimized for F1, and optimized for rule similarity; for each configuration we report precision, recall, F1, and the median and IQR of our three rule similarity metrics.

Significant values are in bold.

Executing rules on claim records to identify non-compliance is a typical classification task: the positive class consists of non-compliant claims. We measured precision ($tp/\left(tp+fp\right)$), recall ($tp/\left(tp+fn\right)$), and F1 (the harmonic mean of precision and recall) for every rule implemented as a ground-truth algorithm ($tp, tn, fn$ are respectively the number of true positive, true negative and false negative outcomes). Clais automatically extracted 18 (90%) of the ground-truth rules corresponding to the algorithms manually developed by domain experts. When executing the 18 automatically extracted rules on the claims database to identify non-compliance, the median precision, recall and F1 of our system were 1.0 (IQR = 0.88 to 1.0), 0.99 (IQR = 0.99 to 1.0), and 1.0 (IQR = 0.83 to 1.0), respectively. We asked the domain experts to use our visual interface (see Fig. 1c) to validate the 18 rules that Clais automatically extracted: 11 rules (61%) did not need any corrections; the remaining seven rules had two types of problems: they either missed a condition, or had one or more incorrect values in an existing condition. Our system achieved F1 1.00 (IQR = 1.0 to 1.0) when executing the validated rules on the claims database ${\mathcal{D}}$.

**Figure 3:**
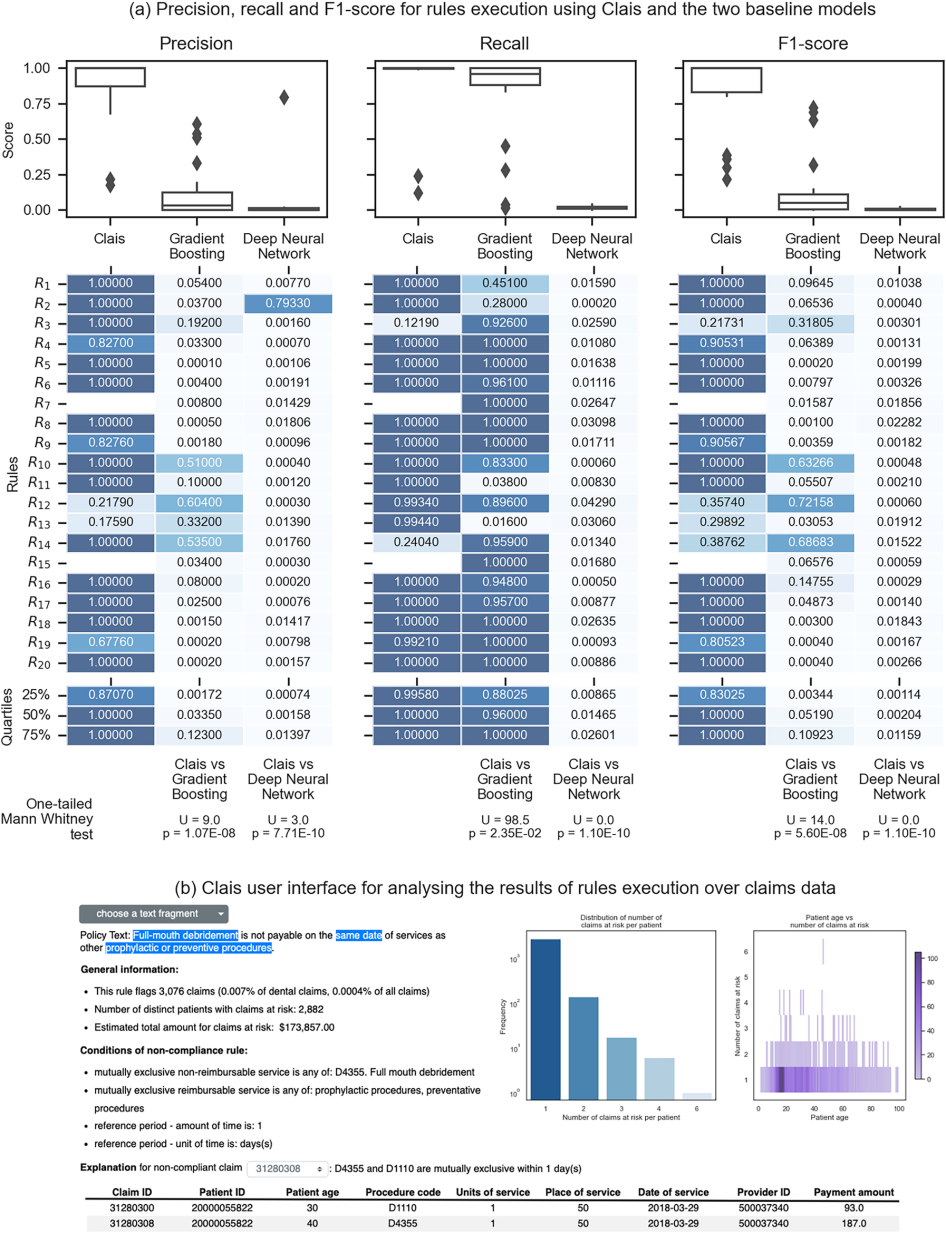
(**a**) Comparison of precision, recall and F1-score for identification of non-compliant claims using Clais and two baseline models (gradient boosting and deep neural network). The baseline models use a subset of the labelled claims data for training, and therefore the evaluation of the three systems is done using a disjoint test-subset of the labelled data. When evaluating Clais we use the automatically extracted rules (as opposed to the same rules curated by human experts); our system does not automatically extract rules ${\text{R}}_{7}$ and ${\text{R}}_{15}$. We also report quartiles of precision, recall and F1-score. The results of one-tailed Mann Whitney test indicate that Clais metrics significantly outperform the baseline models. (**b**) Clais user interface for analysing the results produced by the execution of rules on claims data.

To compare the results of our system with more traditional approaches found in literature, we developed two baseline models: a gradient boosting model and a deep neural network. We split the labelled data for each ground-truth rule into training, validation, and test sets (54%, 6%, and 40% of the data, respectively), and we evaluated Clais and the two baseline models on the same test set. For a fair comparison, we used the automatically extracted rules (instead of rules curated by human experts) when evaluating Clais. Figure 3a compares precision, recall and F1 for Clais and the baseline models. Clais significantly outperformed both the gradient boosting model, and the deep neural network: a one-tailed Mann–Whitney test indicates that precision, recall, and F1 are greater for Clais than for any of the two baseline models.

A distinctive characteristic of our system is its ability to explain the results obtained by executing the rules on claims data (screenshots in Figs. 1f and 3b): explanations are human understandable and include reference to the conditions of the rule extracted from the policy document. The user can choose a fragment of a policy document corresponding to one of the executed rules; the user interface displays aggregated statistics about the claims that the selected rule identified as non-compliant, and a human-friendly summary of the conditions of the rule. Additionally, the user may choose a specific non-compliant claim and see detailed information including data of related claims in the patient history, and a sentence explaining why it is not compliant—for example (Fig. 3b) “D4355 and D1110 are mutually exclusive within 1 day(s)” (D4355 and other similar codes used in claims refer to the procedure codes listed in the Code on Dental Procedures and Nomenclature^38^ defined by the American Dental Association).

Furthermore, we investigated the collaborative aspect of Clais, and specifically its perceived usefulness, with a user study involving 15 participants. All participants were expert policy investigators in the healthcare domain. We divided the participants in two groups: Internal and External. The Internal Group included seven participants who collaborated with us (explaining the domain, the challenges, and the requirements; developing the ground-truth rules, and the 20 ground-truth algorithms). The participants in the Internal Group had the opportunity to use Clais before the user study, for example to define rules, to validate extracted rules, and to analyze potentially non-compliant claims identified by our system. Conversely, the eight participants in the External Group had no exposure to Clais before the user study, except for a one-hour introductory tutorial that we delivered immediately before the user study.

The job role of the participants is either ‘FWA investigator’ or ‘Data Analyst’. Experts from the two job roles typically work together when investigating providers’ claims. FWA investigators often start their work by analyzing a policy document; data analysts typically work more with claims data, either querying them, or writing algorithms to analyze them. The two job roles are almost equally represented in the overall group of participants: seven Data Analysts (2 in the Internal Group and 5 in the External Group) and eight FWA investigators (5 in the Internal Group and 3 in the External Group)..

We investigated the perceived usefulness of our system, which we measured using two well-established standard questionnaires: the PUEU questionnaire^39^, and the USE questionnaire^40^. Both the PUEU and the USE questionnaires use a 7-point Likert scale^41^, where 1 = extremely unlikely, 2 = quite unlikely, 3 = slightly unlikely, 4 = neither unlikely nor likely, 5 = slightly likely, 6 = quite likely, and 7 = extremely likely. The PUEU questionnaire provides a measurement scale for the two variables “perceived usefulness” (PU) (which is defined as “the degree to which a person believes that using a particular system would enhance his or her job performance”), and “perceived ease of use” (EU) (which is defined as “the degree to which a person believes that using a particular system would be free of effort”). The PUEU questionnaire contains 12 questions, 6 for each of the two variables. The USE questionnaire (USE stands for usability, satisfaction, and ease of use) has four sections, measuring usefulness (8 questions), ease of use (11 questions), ease of learning (4 questions) and satisfaction (7 questions). We asked participants of the user study to answer all questions of the USE questionnaire; however, we observe that while the first two sections (usefulness and ease of use) are directly comparable with the respective sections of the PUEU questionnaire, the last two sections (ease of learning and satisfaction) are not comparable with PUEU, and are of less interest for our study, which investigated the perceived usefulness of Clais.

All participants answered all questions of the PUEU questionnaire, and all questions of the usefulness section of the USE questionnaire; we had 4.22% missing answers in the other sections of the USE questionnaire. We decided to assign the neutral score 4 of the 7-point Likert scale to the missing answers, because its meaning (“neither unlikely nor likely”) is semantically consistent with the respondent not providing any answer to the question.

**Figure 4:**
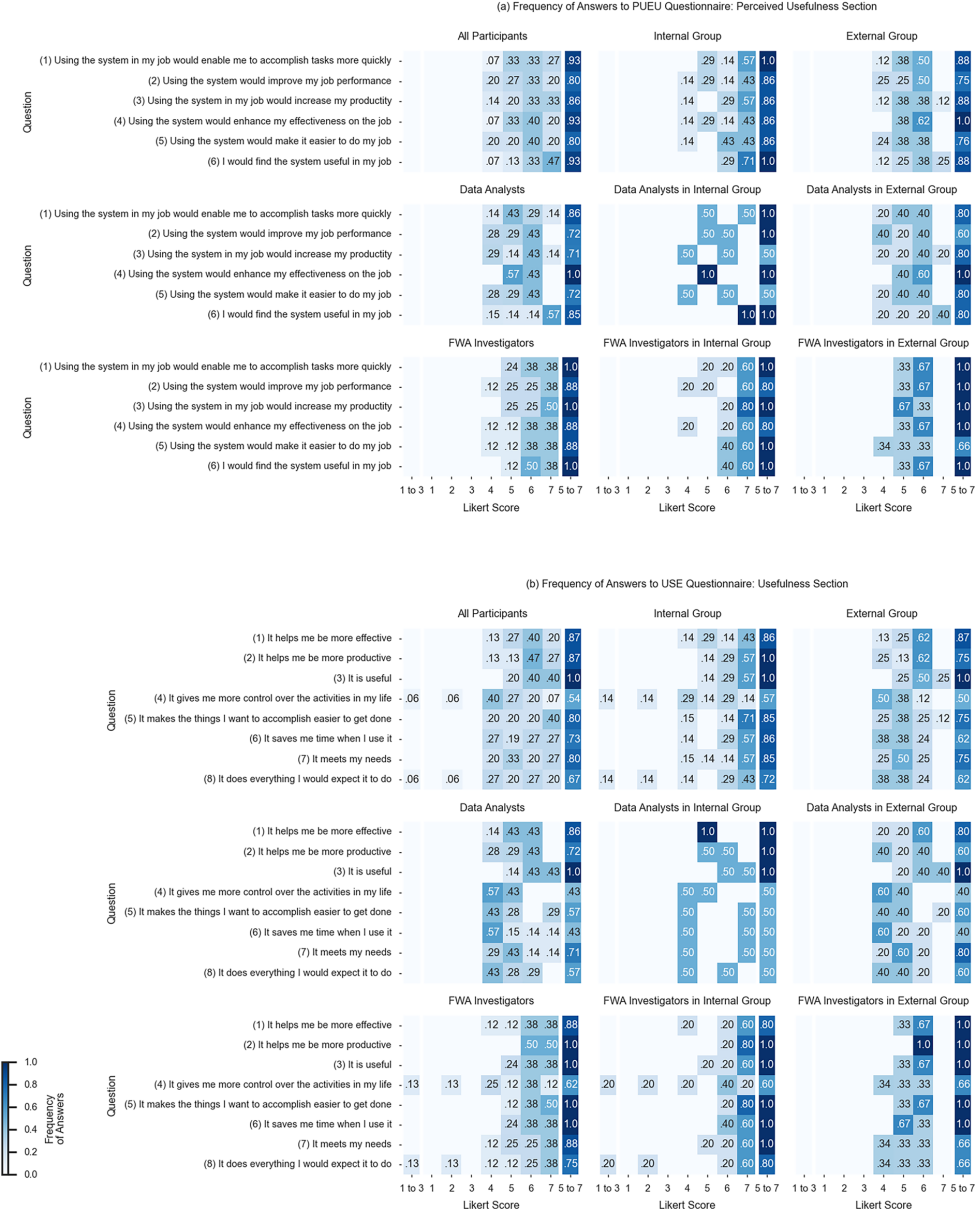
Frequency of answers to (**a**) the perceived usefulness section of the PUEU questionnaire, and (**b**) the usefulness section of the USE questionnaire. The heatmaps show frequencies of responses for different groups of participants. Each heatmap shows the frequency of responses (Likert scores from 1 to 7; only values larger than 0 are reported); at the right of each heatmap we aggregate the overall frequency of positive responses (Likert scores 5, 6, and 7); similarly, at the left of each heatmap we aggregate the overall frequency of negative responses (Likert scores 1, 2, and 3).

Table 2 reports the percentage of participants (also aggregated by group and job role) who answered positively (Likert score 5, 6, or 7) to all the questions related to usefulness—questions 1 to 6 of the PUEU questionnaire (PUEU[1:6]) and questions 1 to 8 of the USE questionnaire (USE[1:8]). No participant answered negatively (Likert score 1, 2, or 3) or neutrally (Likert score 4) to all questions related to usefulness. Figure 4 shows the frequency of answers to the Perceived Usefulness section of the PUEU questionnaire and the Usefulness section of the USE questionnaire. Answers are concentrated in the positive range of the Likert scale (scores 5, 6, and 7).

**Table 2:** Percentage of participants who answered positively (Likert score 5, 6, or 7) to all questions related to usefulness.

	PUEU questionnaire: perceived usefulness section (%)	USE: questionnaire: usefulness section (%)
All participants	60	47
Internal group	71	57
External group	50	38
Data analysts	43	29
FWA investigators	75	62
Data analysts in the internal group	50	50
FWA investigators in the internal group	80	60
Data analysts in the external group	40	20
FWA investigators in the external group	67	67

We analyzed in more detail the answers addressing the usefulness of the system in both questionnaires (PUEU[1:6] and USE[1:8]): we wanted to investigate if the tendency to answer positively to such questions was the same for various subgroups of users who participated in the study, and if positive answers were more likely than negative answers. The categorical variable “answer positively to a questionnaire section” is based on the following assumptions: (a) an answer to a question was positive if its Likert score is greater than 4 (we consider neutral answers as non-positive), (b) a participant answered positively to a questionnaire section if she/he answered positively to more than half of the questions in the section. Given the small sample size, we used a two-tailed Fisher’s exact test with the following pairs of subgroups: (1) “Internal Group and External Group”, (2) “Data Analysts and FWA Investigators”, (3) “Data Analysts in Internal Group and FWA Investigators in Internal Group”, (4) “Data Analysts in External Group and FWA Investigators in External Group”, (5) “Data Analysts in Internal Group and Data Analysts in External Group”, and (6) “FWA Investigators in Internal Group and FWA Investigators in External Group”. Our null hypothesis is that the tendency to answer positively to a questionnaire section is the same for any pair of subgroups. For all 12 cases (the 2 questionnaire sections PUEU[1:6] and USE[1:8], and the 6 pairs of subgroups), we fail to reject the null hypothesis, and therefore we cannot find (given the data collected in our user study) a statistically significant difference in the tendency to answer positively to PUEU[1:6] or USE[1:8] for all pairs of subgroups.

To investigate if positive answers were more likely than negative answers, we made the same assumptions (a) and (b), and additionally, we considered that participants answered independently and without influencing each other (because of how the user study was conducted). Under these assumptions, we used a one-sided binomial test, and our null hypothesis was that no more than 50% of the population answered positively to PUEU[1:6] or USE[1:8]. The data collected in our user study allowed us to reject the null hypothesis when considering all participants, or those having a job role of FWA Investigators (both in the Internal and External Group). More precisely, for PUEU[1:6] a positive answer is significantly more likely than a negative answer when looking at data collected from all professionals (empirical proportion = 0.93, p-value = 0.00049, 95% confidence interval = [0.72, 1.00]), or from FWA Investigators (empirical proportion = 1.0, p-value = 0. 0039, 95% confidence interval = [0.69, 1.00]). For USE[1:8] a positive answer is significantly more likely than a negative answer when looking at data collected from all professionals (empirical proportion = 0.8, p-value = 0.0176, 95% confidence interval = [0.56, 1.00]), or from FWA Investigators (empirical proportion = 1.0, p-value = 0.0039, 95% confidence interval = [0.69, 1.00]).

## Discussion

In this study, we present Clais, a collaborative AI system for claims analysis, which supports the workflow of professionals in healthcare fraud, waste, and abuse, and helps them identify non-compliance in providers’ claims. Clais overcomes limitations of existing systems that use manually defined compliance rules (costly and difficult to maintain) or data-driven machine learning algorithms (requiring large amounts of high-quality labelled data, and possibly lacking interpretability). Clais automatically extracts rules from healthcare policy documents. The rules are human-interpretable: professionals can interact with them in a visual interface enabling modification and validation of the rules. Clais executes the (validated) rules directly on claim records, identifies non-compliant claims, and reports their data, the violated rule(s), the corresponding fragment(s) of policy text, and a human understandable explanation of why a specific claim is not compliant. Clais ability to provide useful and human understandable explanations of its results (confirmed in our user study) is a step forward in the direction of trustworthy artificial intelligence, and specifically in the possibility of achieving trust through counterfactual explanations^42^.

**Figure 5:**
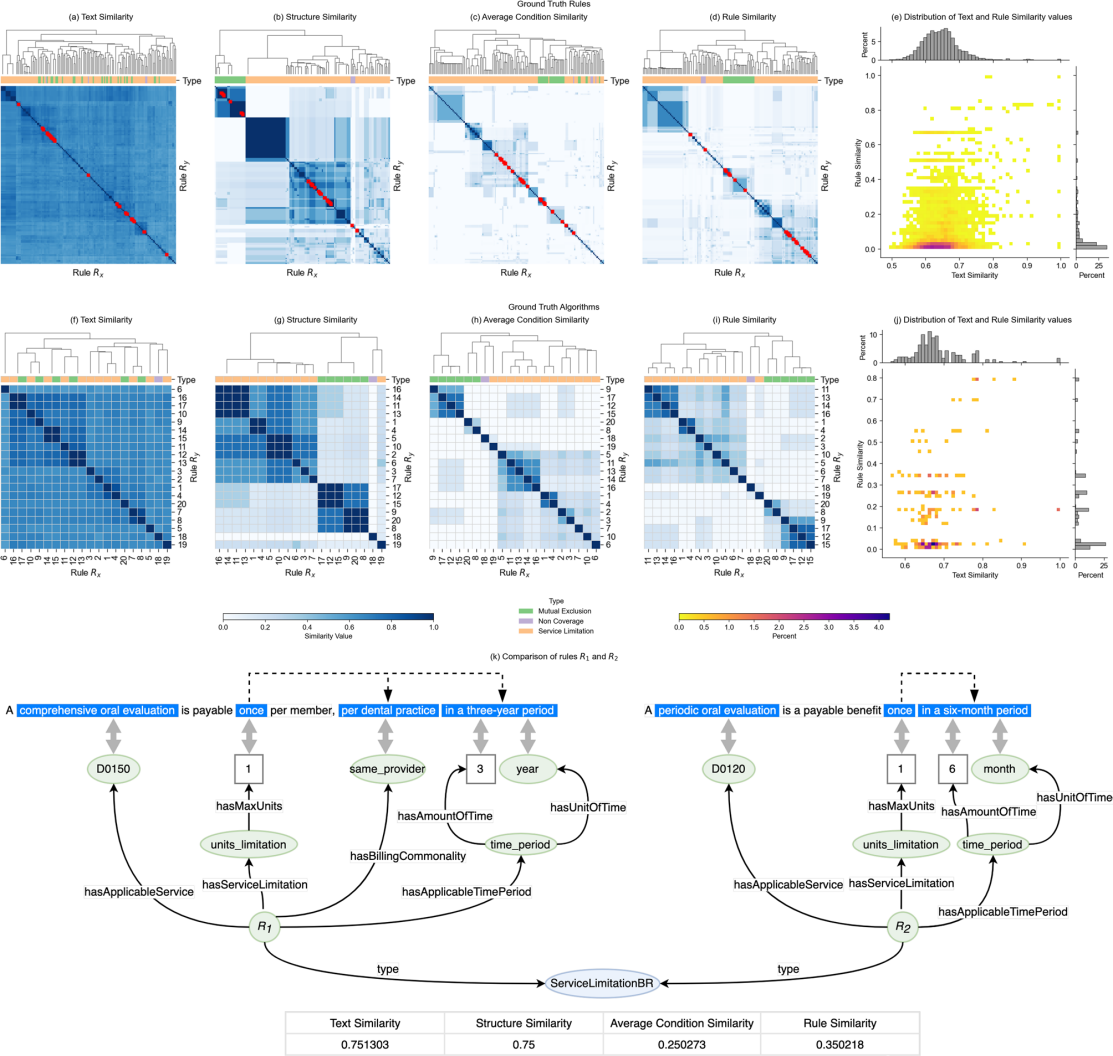
Analysis of the pairwise similarity of the ground truth rules, and the ground truth algorithms. (**a–i**) are hierarchically clustered heatmaps of the text similarity, structure similarity, average condition similarity and overall rule similarity for ground truth rules and ground truth algorithms, respectively. Red markers in (**a–d**) identify ground truth algorithms. The dendrograms show how rules are clustered: we colour the leaves of the dendrograms to show how the clustering algorithm groups rules according to their type (mutual exclusion, non-coverage, and service limitation); for these plots we used seaborn^43^ clustermap function with default method (average) and default metric (Euclidean) to compute the hierarchical clusters. The numbers along the horizontal and vertical axis of (**f–i**) identify the rules corresponding to the ground truth algorithms. The bivariate histograms (**e,j**) show the distributions of the values of the rule similarity and text similarity metrics for every distinct pair ${\text{R}}_{\text{x}},{\text{R}}_{\text{y}}$ ($\text{x}\ne \text{y}$)—(**e**) shows the distributions for ground truth rules, and (**j**) for ground truth algorithms. The similarity metrics are symmetric, and therefore we consider only distinct pairs: if we consider $\left({\text{R}}_{\text{x}},{\text{R}}_{\text{y}}\right)$ then we omit $\left({\text{R}}_{\text{y}},{\text{R}}_{\text{x}}\right)$. The bivariate histograms show the distribution of the values of two similarity metrics by tiling the data space $[0, 1] \times [0, 1]$ with 2500 bins. The color of the bivariate histograms shows the percentage of observations in each bin. The marginal histograms show the distributions of text similarity (horizontal marginal) and the rule similarity (vertical marginal); both marginal histograms tile the respective data space $[0, 1]$ with 50 bins, and the height of the bar is proportional to the percentage of observation in each bin. We used seaborn^43^ jointplot function to plot the bivariate histograms with marginals. (**k**) Compares in more details two ground truth algorithms (${\text{R}}_{1}\text{ and}{\text{ R}}_{2}$); this is a typical example of two rules having high text similarity, but low rule similarity.

The automatic extraction of executable rules from policy text is a complex task. To the best of our knowledge, there is no prior work in the healthcare domain addressing this task, and there is no available ground-truth data for building or evaluating systems. We contribute as open source a set of ground truth rules and a related ontology^32^. The findings of this study suggest that Clais is effective at automatically extracting (partially) correct rules from policy documents, and complements recent work in the area of ‘Rules As Code’^4–7^ showing that an AI system can collaborate with humans to create machine-consumable and human-understandable rules to accompany existing natural language policy documents. This study and our system have some limitations. Firstly, we tested Clais on policy documents in the dental domain: extension to other domains requires adapting the ontology that guides the extraction of rules from text, and possibly fine tuning the extraction pipeline to recognize domain-specific entities from text (other components of Clais are domain-independent). Secondly, the ground truth rules that we developed may not be an exhaustive set, and future work includes developing a more extensive one. Nevertheless, we observe that our ground truth rules are very diverse: Fig. 5 compares four similarity metrics for all ground truth rules and for the 20 rules corresponding to the ground truth algorithms. Considering all ground truth rules, the median text similarity is 0.64 (IQR = 0.61 to 0.67), the median structure similarity is 0.2 (IQR = 0.13 to 0.4), the median for the average condition similarity is 0 (IQR = 0 to 0.04), and the median rule similarity is 0.03 (IQR = 0.01 to 0.14). Ground truth rules are quite similar from a textual point of view (the text similarity is the angular similarity between embedding vectors encoding the texts); the bivariate histogram in Fig. 5e shows that most rules have text similarity between 0.6 and 0.7 but a rule similarity very close to 0. The relatively high text similarity is not surprising: text fragments describe compliance and non-compliance regulations in the dental domain, and therefore the variations in terminology and sentence structure are small. Additionally, two rules (typically a service limitation rule and a rule describing services that are mutually exclusive) may often refer to the same text fragment. Figure 5k shows a typical example of two ground truth rules having high text similarity (0.75) but low rule similarity (0.35): the structure similarity is high (both rules define service limitations and have similar conditions), but the values of the conditions (even common ones) are very different, and therefore the average condition similarity and the overall rule similarity are low. Although sentences describing our ground truth rules are relatively similar, we observe that the metrics measuring their logical similarity (structure, conditions, and overall rule similarity) are very low: considering all distinct pairs of ground truth rules having a text similarity greater than the median value (0.64), only 4% have a rule similarity greater than 0.5, and only 1% have a rule similarity greater than 0.7: this shows a considerable diversity of the logical fabric of our ground truth rules. The rules corresponding to the ground truth algorithm are also largely diverse: the median text similarity is 0.67 (IQR = 0.65 to 0.72), the median structure similarity is 0.33 (IQR = 0.17 to 0.75), the median for the average condition similarity is 0.10 (IQR = 0.00 to 0.21), and the median rule similarity is 0.17 (IQR = 0.02 to 0.29); considering all distinct pairs of ground truth algorithms having a text similarity greater than the median value (0.67), only 14% have a rule similarity greater than 0.5, and only 6% have a rule similarity greater than 0.7. The diversity of our ground truth helps to support our findings related to the performance of Clais in automatically extracting rules from text and in executing the rules to identify non-compliant claims. Further studies may extend our ground truth to evaluate performance in other medical domains.

Previous work^15,17,21^ has used machine learning and deep learning to identify fraudulent claims and providers in public datasets. However, existing methods do not consider policy documents, but only historical claims that have been labelled as fraudulent. Our approach is different. Firstly, it does not require training on historical labelled data, but it can identify potentially non-compliant claims even without access to previous (similar) samples. Secondly, it promotes standardization and re-usability by formalizing policy text into structured rules that are both human-interpretable and machine-executable. We cannot directly compare our approach with previous work^15,17,21^, because their datasets do not include the policy documents that Clais requires to extract rules and do not provide compliance information at the granularity of a single claim (which is what we aim to detect with our system) but only at provider level. For these reasons we trained two baseline models (a gradient boosting model and a deep neural network) on the same dataset of labelled claims that we use to execute the rules extracted by Clais. Our findings show that our system significantly outperforms both baseline models (see Fig. 3). There are two cases (see rules ${R}_{12}$ and ${R}_{14}$ in Fig. 3) in which the gradient boosting model exhibits a better F1-score than Clais. Rule ${R}_{12}$ corresponds to the following policy text: “an oral evaluation for children under three years of age and counseling with the primary caregiver (D0145) is payable once every six months”. Our system misinterprets the expression “under three years” and assigns the value “3” to the rule condition “hasMaxAge”, thus including patients of three years of age: this error has a negative impact on precision when executing the rule. Rule ${R}_{14}$ also has a problem with a value that is not correctly extracted from the following text: “complete and partial dentures are payable once in a five-year period”. The text describes a service limitation on “complete dentures” and “partial dentures”; the automatically extracted rule contains the condition “hasApplicableService” to express the service limitation, but its value includes only “partial dentures”. This error has a negative impact on recall when executing the rule. For both rules ${R}_{12}$ and ${R}_{14}$, Clais uses correct conditions but fails to extract correct values; this kind of errors is easy to identify and correct by human experts when validating the rules using Clais user interface.

In general, we observe that gradient boosting models have poor performance because most rules need some aggregation of historical claims data to verify compliance. Such aggregations require manual feature engineering for each specific rule, with an implementation effort comparable to direct implementation of rule algorithms by policy experts. The deep neural network, which we implemented as a recurrent neural network^44–46^ to capture the temporal nature of historical claims data, also has very poor performance. Besides the well-known problems of this type of network (vanishing or exploding gradient^47^), we observe that rules defined in policy documents usually have different temporal aggregations (ranging from months to years). We speculate that such variety of temporal aggregations makes training of the neural network difficult, and unstable, thus causing generalization problems which prevents the network from learning useful patterns.

Finally, the findings in this study confirm that professionals perceive Clais as useful to support their work. We observe that the tendency to answer positively to the usefulness sections of the PUEU and USE questionnaires is predominant for the job role FWA Investigators. This suggests that the design and development of Clais has been influenced by professionals having this job role (71% of the experts in the Internal Group are FWA Investigators). In accordance with the previous observation, we note that Data Analysts in the External Group provided comments to their answers in the user study suggesting additional features related to the exploration of claims data (for example, they asked to see the queries run by our system to identify claims at risk for a given rule, or the mapping between the conditions of a rule and the fields of the claims database). Such comments are very useful to plan future development of Clais.

## Method

### Formal definition of rule and similarity metrics

A rule is a logical representation of a section of text in a policy document. We formally define a rule $R={\bigwedge }_{i=1}^{n}{C}_{i}$ as the conjunction of the set of conditions $\mathcal{C}\left(R\right)=\{{C}_{1},{C}_{2},\dots ,{C}_{n}\}$, where each condition ${C}_{i}$ is defined by a property in our ontology, for example “hasMinAge” or “hasExcludedService”. The ontology also specifies restrictions on properties such as cardinality or expected data types. We refer to the set of values of condition ${C}_{i}$ in rule $R$ as $\mathcal{V}\left({C}_{i},R\right)=\{{v}_{1},{v}_{2},\dots ,{v}_{m}\}$; multiple values are interpreted as a disjunction. If $\mathcal{V}\left({C}_{i},R\right)$ contains only one numeric value (for example “hasMinAge(12)”), then we refer to ${C}_{i}$ as a numeric condition.

Given two rules ${R}_{x}$ and ${R}_{y}$, and a condition ${C}_{i}$, we define the condition similarity metric as:

$${\mathcal{S}}_{C}\left({C}_{i},{R}_{x},{R}_{y}\right)= \left\{\begin{array}{cc}\frac{1}{1+\left|{v}_{i, x} - {v}_{i, y}\right|}& \begin{array}{c}if \,{C}_{i}\in \mathcal{C}\left({R}_{x}\right) \;and \,{C}_{i}\in \mathcal{C}\left({R}_{y}\right)\; and \,{C}_{i} \,is\, numeric\\ and \; \mathcal{V}\left({C}_{i},{R}_{x}\right)=\{{v}_{i, x}\}\; and \;\mathcal{V}\left({C}_{i},{R}_{y}\right)=\{{v}_{i, y}\}\end{array}\\ \frac{\left|\mathcal{V}\left({C}_{i},{R}_{x}\right)\cap \mathcal{V}\left({C}_{i},{R}_{y}\right)\right|}{\left|\mathcal{V}\left({C}_{i},{R}_{x}\right)\cup \mathcal{V}\left({C}_{i},{R}_{y}\right)\right|}& \text{if }{C}_{i}\in \mathcal{C}\left({R}_{x}\right)\text{ and }{C}_{i}\in \mathcal{C}\left({R}_{y}\right)\text{ and }{C}_{i}\text{ is not numeric}\\ 0& \text{ otherwise}\end{array}\right.$$

If ${C}_{i}$ is a numeric condition, and is present in both ${R}_{x}$ and ${R}_{y}$, then the condition similarity ${\mathcal{S}}_{C}\left({C}_{i},{R}_{x},{R}_{y}\right)$ is inversely proportional to the distance between ${v}_{i, x}$ (the numeric value of ${C}_{i}$ in ${R}_{x}$) and ${v}_{i, y}$ (the numeric value of ${C}_{i}$ in ${R}_{y}$). If instead, ${C}_{i}$ is present in both ${R}_{x}$ and ${R}_{y}$, but it is not numeric, then the condition similarity ${\mathcal{S}}_{C}\left({C}_{i},{R}_{x},{R}_{y}\right)$ is the Jaccard similarity^48^ between the set of values of ${C}_{i}$ in ${R}_{x}$ and the set of values of ${C}_{i}$ in ${R}_{y}$. Finally, when ${C}_{i}$ is missing in either ${R}_{x}$ or ${R}_{y}$, the condition similarity ${\mathcal{S}}_{C}\left({C}_{i},{R}_{x},{R}_{y}\right)$ is equal to 0.

Given two rules ${R}_{x}$ and ${R}_{y}$, we also define the rule structure similarity as the Jaccard similarity between the set of conditions in ${R}_{x}$ and the set of conditions in ${R}_{y}$:

$${\mathcal{S}}_{S}\left({R}_{x},{R}_{y}\right)= \frac{\left|\mathcal{C}\left({R}_{x}\right)\cap \mathcal{C}\left({R}_{y}\right)\right|}{\left|\mathcal{C}\left({R}_{x}\right)\cup \mathcal{C}\left({R}_{y}\right)\right|}$$

When ${R}_{x}$ is a ground-truth rule and ${R}_{y}$ is automatically extracted from the same paragraph of text corresponding to ${R}_{x}$, then we use a slightly modified version of condition similarity and structure similarity, where we replace the Jaccard similarity between the set of values and the set of conditions with the Sørensen–Dice coefficient^36,37^ between the same sets. More precisely:

$${\mathcal{S}}_{C}\left({C}_{i},{R}_{x},{R}_{y}\right)= \left\{\begin{array}{cc}\frac{1}{1+\left|{v}_{i, x} - {v}_{i, y}\right|}& \begin{array}{c}if \;{C}_{i}\in \mathcal{C}\left({R}_{x}\right) \;and \;{C}_{i}\in \mathcal{C}\left({R}_{y}\right) \;and \;{C}_{i} \;is \;numeric\\ and \;\mathcal{V}\left({C}_{i},{R}_{x}\right)=\{{v}_{i, x}\}\; and \;\mathcal{V}\left({C}_{i},{R}_{y}\right)=\{{v}_{i, y}\}\end{array}\\ \frac{2\cdot \left|\mathcal{V}\left({C}_{i},{R}_{x}\right)\cap \mathcal{V}\left({C}_{i},{R}_{y}\right)\right|}{\left|\mathcal{V}\left({C}_{i},{R}_{x}\right)\right| + \left|\mathcal{V}\left({C}_{i},{R}_{x}\right)\right|}& \text{if }{C}_{i}\in \mathcal{C}\left({R}_{x}\right)\text{ and }{C}_{i}\in \mathcal{C}\left({R}_{y}\right)\text{ and }{C}_{i}\text{ is not numeric}\\ 0& \text{ otherwise}\end{array}\right.$$

$${\mathcal{S}}_{S}\left({R}_{x},{R}_{y}\right)= \frac{2\cdot \left|\mathcal{C}\left({R}_{x}\right)\cap \mathcal{C}\left({R}_{y}\right)\right|}{\left|\mathcal{C}\left({R}_{x}\right)\right| + \left|\mathcal{C}\left({R}_{x}\right)\right|}$$

The Sørensen–Dice coefficient gives a better measure of the accuracy of the system when extracting rules from text. When ${R}_{x}$ is a ground-truth rule and ${R}_{y}$ is the corresponding automatically extracted rule, we consider $\mathcal{C}\left({R}_{x}\right)$ and $\mathcal{C}\left({R}_{y}\right)$ as, respectively, the set of expected and predicted conditions in the rule, and $\mathcal{V}\left({C}_{i},{R}_{x}\right)$ and $\mathcal{V}\left({C}_{i},{R}_{y}\right)$ as, respectively, the set of expected and predicted values for condition ${C}_{i}$ in the rule. In this scenario the Sørensen–Dice coefficient is equivalent to the F1-score, and it measures the harmonic mean of the precision and recall of Clais when extracting ${R}_{y}$.

Finally, we define the overall rule similarity ${\mathcal{S}}_{R}\left({R}_{x},{R}_{y}\right)$ and the text similarity ${\mathcal{S}}_{T}\left({R}_{x},{R}_{y}\right)$ between rules ${R}_{x}$ and ${R}_{y}$ as follows:

$${\mathcal{S}}_{R}\left({R}_{x},{R}_{y}\right)= \frac{{\mathcal{S}}_{S}\left({R}_{x},{R}_{y}\right)+\sum_{{C}_{i}\in \mathcal{C}\left({R}_{x}\right)\cup \mathcal{C}\left({R}_{y}\right)}{\mathcal{S}}_{C}\left({C}_{i},{R}_{x},{R}_{y}\right)}{1+\left|\mathcal{C}\left({R}_{x}\right)\cup \mathcal{C}\left({R}_{y}\right)\right|}$$

$${\mathcal{S}}_{T}\left({R}_{x},{R}_{y}\right) = 1 - \frac{arccos\left(\frac{{u}_{x}\cdot {u}_{y}}{\Vert {u}_{x}\Vert \Vert {u}_{y}\Vert }\right)}{\pi }$$

The overall rule similarity is the arithmetic mean of the structure similarity ${\mathcal{S}}_{S}\left({R}_{x},{R}_{y}\right)$ and the condition similarity ${\mathcal{S}}_{C}\left({C}_{i},{R}_{x},{R}_{y}\right)$ for all conditions ${C}_{i}$ in ${R}_{x}$ or ${R}_{y}$. The text similarity ${\mathcal{S}}_{T}\left({R}_{x},{R}_{y}\right)$ is the angular similarity between the embedding vectors ${u}_{x}$ and ${u}_{y}$ encoding the sections of text corresponding to ${R}_{x}$ and ${R}_{y},$ respectively. We use Sentence-BERT (SBERT)^49^ with the state of the art model “all-mpnet-base-v2”^50^ to compute the embedding vectors; similar to other work^51^, we convert the cosine similarity between the embedding vectors into an angular distance in the range [0, 1] using arccos and normalizing by $\pi$.

### Extraction of rules from text

Clais uses knowledge graphs to represent rules. A knowledge graph is a directed labelled graph (example in Fig. 1d), and we encode its structure and semantics using RDF^52^ triples (subject, predicate, object). Our ontology^32^ (excerpt in Fig. 1b), designed in collaboration with expert policy investigators^25^, formally defines the meaning of subjects, predicates and objects used in the rule knowledge graphs. The ontology also specifies restrictions on the predicates (for example, expected domain and range, disjoint or cardinality constraints), which guide our system in building semantically valid RDF triples and meaningful rules. Additionally, the ontology defines rule types, which consist of concepts-relationships templates capturing repeatable linguistic patterns in policy documents. The current version of the ontology specifies three rule types: (1) limitations on services, such as units of service or reimbursable monetary amounts that a provider can report for a single beneficiary over a given period; (2) mutually exclusive procedures that cannot be billed together for the same patient over a period; and (3) services not covered by a policy under certain conditions.

The ontology design ensures that every rule knowledge graph can be modelled as a tree (an undirected graph in which any two vertices are connected by exactly one path), where leaves are values of the conditions in the rule. The tree representation enables Clais to visualize the rule’s conditions and their values in an intuitive user interface, which simplifies editing and validation of the rule. The same user interface also supports the interactive creation of new rules: professionals compose a rule by selecting items from a library of conditions based on the property defined in the ontology; the system asks for condition values and checks their validity in accordance with the restrictions defined for the corresponding ontology predicate (for example domain, range, cardinality).

**Figure 6:**
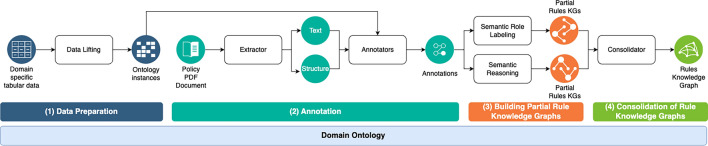
Clais NLP extraction pipeline, which transform a policy PDF document and related domain-specific tabular data into a knowledge graph describing the policy rules. The four stages of the NLP extraction pipeline rely on the general domain ontology.

We build upon recent natural language processing (NLP) techniques^24,25^ to automatically identify dependencies between relevant entities and relations described in a fragment of policy text, and to assemble them into a rule. Clais uses a configurable NLP extraction pipeline, where each component can be replaced or complemented by others with similar functionalities. The configuration can be customized either manually or using hyper-parameter optimization^53,54^ to tune the overall performance of the extraction pipeline for a given policy, domain or geographic region (specifically, we use the Optuna^55^ hyperparameter optimization framework). Clais NLP extraction pipeline (Fig. 6), which does not require labelled data, consists of the following steps: (1) data preparation according to the policy domain and geography (state/region); (2) automatic annotation of policy text fragments to identify mentions of domain entities and relations in the text; (3) building of rule knowledge graphs corresponding to policy text segments using their domain entities and relations in accordance to the ontology definitions; (4) knowledge graph consolidation and filtering to produce a set of well-formed rules (necessary when different components/approaches are used to build the knowledge graphs, or when rules extend across multiple sentences in the policy text).

We automated the data preparation step with tools that use configurable mappings to translate any existing tabular data (used to define domain specific values for a target policy in a geographical region) into ontology instance values of a specific entity type. The tabular data sources contain both the surface forms (meaningful textual labels, synonyms, etc.) necessary to identify mentions in the policy text, and their mapping to ontological resources. The system uses the surface forms for entity and relation annotation in the cold-start scenario (lack of labelled data); the surface forms are usually part of existent tabular data, such as the ones describing codes for eligible places of service^56^ (“hospital”, “clinic”, etc.) or the Healthcare Common Procedure Coding System (HCPCS)^57^ published by Centers for Medicare and Medicaid.

We use state of the art PDF tools to extract the text and the structure of a policy document. The annotation of text relies on ontology-based annotators^58^ using dictionary-based lemma matches. In addition, we initialize the annotators with entity labels such as UMLS types^59^, which are useful to fill in values for some ontology properties (depending on their range definition).

The core of the NLP extraction pipeline transforms textual patterns between ontological annotations into sets of RDF triples, which constitute a partial rule knowledge graph; it uses two domain-agnostic approaches: semantic role labeling^25^ and semantic reasoning^24^. Semantic role labeling is based on a declarative information extraction system^58^ that can identify actions and roles in a sentence (who is the agent, what is the theme or context of the action, if there are any conditionals, the polarity of the action, temporal information, etc.). We use the ontology definitions for the domain and range of properties to reason over the semantic roles, and to identify semantically compatible relation and entity/value pairs in a sentence. Semantic reasoning transforms syntactic dependencies from a dependency tree^60,61^ into RDF triples. Dependency trees capture fine-grained and relevant distant (not necessarily consecutive in the text) syntactic connections by following the shortest path between previously annotated ontological entities. For each linguistically connected predicate-arguments tuple in the sentence, the dependency tree searches the ontology definitions for non-ambiguous paths that could connect the tuple elements in a semantically correct way; the search is based on parametrized templates^24^.

Semantic role labelling and semantic parsing may produce partially overlapping knowledge graphs from the same paragraph of text: the final stage of the extraction pipeline^24^ consolidates them into one final rule knowledge graph, and it also filters potential inaccuracies, based on heuristics that detect violations of ontological constraints such as disjointness (a procedure code cannot be both reimbursable and non-reimbursable in the same rule) or cardinality restrictions (there can only be one applicable ‘time period’ for each rule).

We observe that the extraction pipeline does not require training with labelled data. However, as users validate policy rules, they progressively build a reusable, shared library of machine-readable and labelled rules, which Clais gradually incorporates into two deep learning models^26^ (based on BERT—Bidirectional Encoder Representations from Transformers^62^) that complement the other components of the extraction pipeline: a classifier that identifies paragraphs of text potentially containing a rule description in new policy documents, and a model that predicts the probability that a text fragment provides conditions (and values) for a rule.

### Execution of rules on claims

Clais executes rules on claims data through dynamically constructed software pipelines whose components translate the semantics of rule conditions into executable code. The workflow consists of three stages: first, the system normalizes the conditions of a rule $R$ into a format amenable to execution; second, it transforms the normalized conditions into an evaluation pipeline ${P}_{R}$ by assembling executable components; and third, it executes instances of ${P}_{R}$ and reports the results.

Rules automatically extracted from a policy document (or manually defined by professionals) may be either compliance rules (defining conditions characterizing valid claims) or non-compliance rules (defining conditions that identify claims at-risk). When analyzing claims data, the typical task consists of identifying non-compliant claims, and therefore Clais executes only non-compliant rules. The system transforms a compliance rule into a non-compliance rule by changing one (or more) of its conditions according to their semantics (ontology definitions), and to the logic structure of the rule (logical negation). Figure 1c–e show an example of such transformation: the policy text “A periodic oral evaluation is a payable benefit once in a six-month period” is a compliance rule defining a unit limitation: “it is compliant to claim at most one unit of service within six months”. The corresponding knowledge graph models the unit limitation using the conditions “hasMaxUnits(1)” (for brevity we omit conditions defining the temporal aspect of the rule). Clais transforms the rule into a non-compliance rule by changing “hasMaxUnits(1)” into “hasMinUnit(2)”: “it is not compliant to claim two or more units of service within six months”. The preliminary transformation stage may also replace a subset of conditions with a single condition whose semantics maps directly to one of the executable components in the downstream execution pipeline. An example of such transformation is the following: rules typically have some temporal constraints, which are expressed with two conditions: one defining the amount of time (“hasAmountOfTime”), and one defining the unit of time (“hasUnitOfTime”)—see examples in Fig. 1d and Fig. 5k. This pair of conditions is replaced with a single condition that defines a time window in days (“hasSlidingWindowInDays”)—see Fig. 1e.

Clais dynamically assembles an evaluation pipeline for each rule by chaining executable components that perform operations on an evaluation context; the evaluation context consists of the temporal sequence of claim records belonging to a patient. Clais uses three types of executable components: filter, splitter, and evaluator; these are sufficient to map the semantics of the ontology properties that define normalized rule conditions. Future extensions of the ontology may require new types of executable components. The definition of filters, splitters, and evaluators uses declarative functions that query the fields of a claim record and produce some output value; we support the modular expression language SpEL^63^ for writing such functions. Filters apply logical conditions to limit the evaluation context only to claim records that are relevant for the rule being executed. A typical example of a condition that maps to a filter is “hasApplicableService” (Fig. 1e), which restricts the evaluation context to those claims referring to a specific service code. Splitters divide an evaluation context into a stream of evaluation contexts depending on values in the claim records, or on temporal windows; they may also perform grouping operations and compute aggregation on claim records. The condition “hasBillingCommonality(same_provider)” (see Rule ${R}_{1}$ in Fig. 5k) maps to a splitter that groups claim records by their provider identifier; the condition “hasSlidingWindowInDays(180)” (Fig. 1e) maps to a splitter that divides the sequence of claim records into sub-sequences whose temporal duration spans at most 180 days. Finally, an evaluator analyses each claim in an evaluation context using an expression that evaluates to true or false; a typical evaluator produces a true value when the cumulative sum of a specified claim field, over the claims in the evaluation context, is greater (lower) than a given threshold.

Clais uses a configurable mapping to transform conditions in a rule into their respective executable components, and to assemble them in an evaluation pipeline. In each pipeline, the final evaluator component evaluates each claim in its respective evaluation context. A claim may appear in several evaluation contexts depending on the rule conditions: the claim is non-compliant if the evaluator associates a true value with the claim in at least one evaluation context. Clais builds an evaluation pipeline ${P}_{R}$ for every rule $R$; it deploys an instance of ${P}_{R}$ for each patient (the input consists of an evaluation context listing the claims of the patient in chronological order). Each instance of ${P}_{R}$ is executed in parallel: the parallel execution of evaluation pipelines (for different rules and for different patients) can be distributed across a computing infrastructure, thus enabling the scalability required to efficiently process very large volumes of claims (distributed execution was not necessary in our experiments).

### Baseline models

We used a dataset of 44,913,580 dental claims to train our baseline models; 421,321 (0.94%) claims are labelled as fraudulent. In our experiments we used random under-sampling for each rule data with 80:20 distribution (80% normal and 20% fraudulent claims) to build the training dataset (same as previous work^15^). We experimented with several strategies to cope with the problem of class imbalance, including considering different balancing ratios for the dataset and re-weighting of classes in the negative log likelihood loss; the best performance was obtained with a simple random under-sampling with 80:20 distribution.

For both baseline models, we did not perform feature engineering to create additional features, because manual feature engineering is data-dependant. Different rules require different types of features and creating manual features for every rule is impractical, and as costly as manually implementing algorithms for compliance checks (similarly to what policy investigators currently do).

We used the open-source LightGBM^64^ implementation of gradient boosting trees with the following default hyper-parameters: n_estimator = 20,000, max_depth = 6, num_leaves = 1000, learning_rate = 0.01, bagging_fraction = 0.8, min_data_in_leaf = 5. For gradient boosting models, we have rebalanced the data with different balancing ratios ranging from 0.1 to 1.0 but we did not observe an improvement over the reported results (Fig. 3a).

The deep neural network baseline uses a Recurrent Neural Network (RNN) architecture^44–46^ to learn dependencies between elements in a sequence. The sequence used to classify a claim consists of all the preceding claims and related features in chronological order, in addition to the claim being classified. Each claim in the sequence also contains the time-delta from the previous claims. All categorical features are processed with an embedding layer, having a size equal to the square root of the feature cardinality; all numerical features are processed by a normalization layer that estimates the variance and the average during the training phase. The RNN implementation relies on a Gated Recurrent Unit^46^ with 3 hidden layers, trained with a learning rate of 10e-3 using negative log likelihood loss. We use a binary softmax layer as a final claims classification layer. We trained the network for up to 50 epochs (similarly to previous work^21^), but we did not see improvements.

### User study

We organized the user study in two sessions: the first with the External Group (participants had no prior knowledge of Clais), and the second with the Internal Group (participants were also involved in the design and prototyping of Clais and in the development of the ground truth). Each session was organized as a video-conference: each participant used her/his own computer and answered the questionnaires independently. We started each session describing the purpose of the user study, the structure of PUEU and USE questionnaires, and their scope in the context of our user study. The session with the External Group included a one-hour introductory tutorial about Clais. We delivered the questionnaires on spreadsheets; we went through the questions one by one, providing clarifications if necessary, and waited for each participant to answer a question (including writing comments) before moving to the next. We collected the spreadsheets with the answers and removed any reference to the participants’ identity (except for job role and group) before processing the results.

## Conclusions

In this study, we present Clais, a collaborative AI system that helps healthcare investigators to identify non-compliance in providers’ claims. Clais automatically extracts human interpretable rules from healthcare policy documents; it executes the rules directly on claim records, identifies non-compliant claims (on this task Clais significantly outperforms two baseline machine learning models), and provides a human understandable explanation of why a specific claim is not compliant. A user study with professional healthcare investigators confirms the usefulness of Clais in making their workflow simpler and more effective. A distinctive characteristic of our system is its ability to explain the results obtained by executing the rules on claims data, and our user study shows that this is a step forward in the direction of trustworthy AI. Future work includes both testing Clais on healthcare policies outside the dental domain (which may require extensions to the ontology and ground truth presented in this study), further develop the functionality of the system to incorporate suggestions collected during the user study and extend explanations and interpretability of results to further increase trust in the AI system.

## Data Availability

The ontology and ground truth rules generated and/or analysed during the current study (extraction of rules from text) are available in the github repository https://github.com/IBM/rules_extraction_from_healthcare_policy. The (labelled) claim records generated and/or analysed during the current study (execution of rules on claims) are not publicly available due to commercial licenses of the MarketScan database (IBM Watson-Health/Merative https://www.merative.com/documents/brief/marketscan-explainer-general); such data are however available from the authors upon reasonable request and with permission of IBM Watson-Health/Merative. Finally, The anonymized responses collected during the user study are available from the corresponding author upon reasonable request.
